# Potential Role of microRNA-21 in the Diagnosis of Gastric Cancer: A Meta-Analysis

**DOI:** 10.1371/journal.pone.0073278

**Published:** 2013-09-04

**Authors:** Zongyue Zeng, Jiangen Wang, Liuyang Zhao, Ping Hu, Hailong Zhang, Xi Tang, Dali He, Shifu Tang, Zhaofang Zeng

**Affiliations:** College of Laboratory Medicine, Key Laboratory of Laboratory Medical Diagnostics designated by Chinese Ministry of Education, Chong Qing Medical University, Chongqing, China; Pontificia Universidad Catolica de Chile, Faculty of Medicine, Chile

## Abstract

**Introduction:**

Accumulating evidences indicate that microRNA-21(miR-21) show significant high concentration in plasma of gastric cancer (GC) patients compared to normal individuals, suggesting that it may be a useful novel diagnostic biomarker for gastric cancer. Therefore, we aimed to assess the potential diagnostic value of miR-21 for gastric cancer in this study.

**Methods:**

Literature database including PubMed, Embase, the Cochrane Library, Web of Science, Ovid, SciVerse, Science Direct, Scopus, BioMed Central, Biosis previews,Chinese Biomedical Literature Database (CBM), Chinese National Knowledge Infrastructure (CNKI), Technology of Chongqing (VIP), and Wan Fang DATA were searched for publications concerning the diagnostic value of miR-21 for GC without language restriction. The quality of each study was scored with the Quality Assessment of Diagnostic Accuracy Studies (QUADAS). Then, data were retrieved from any qualified article hits and subject to meta-analysis. Receiver operating characteristic curves (ROC) were used to check the overall test performance. Evidence of heterogeneity was evaluated using the Chi-square and *I* ^2^ test.

**Results:**

Five studies with a total 251 GC patients and 184 control individuals were included in this meta-analysis. All of the included studies are of high quality (QUADAS score$13). The summary estimates revealed that the pooled sensitivity is 66.5% (95% confidence interval (CI): 55.0%–76.3%) and the specificity is 83.1% (95% CI: 69.4%–91.5%). In addition, the area under the summary ROC curve (AUC) is 0.80.

**Conclusion:**

The current evidence suggests that miR-21 has potential diagnostic value with a moderate sensitivity and specificity for GC. More prospective studies on the diagnostic value of miR-21 for GC are needed in the future.

## Introduction

Gastric cancer is the fourth commonest malignant tumor in the world, and it is also the third leading cause of cancer death in men and the fifth leading cause in women [1]. Furthermore, less than 25% of GC cases are diagnosed at the early stage, and the 5-year survival rate is only 26% in the United States, 20%–25% in the Europe and China [1]–[3]. However, the survival rate for GC can increase to more than63% [1]. Since the prognosis of GC is closely related to the extent that how early the disease is diagnosed and subjected to proper treatment, efficient diagnostic methods and effective therapeutic strategy are urgently needed in clinic GC medical care process. Currently, large part of the efforts focuses on identification of serum biomarkers for GC [4]. However, on the basis of current evidence, there are few reliable biomarkers for the diagnosis of GC. Some reported biomarkers (for example, pepsinogens I and II, gastrin-17, interleukin-8, antibodies against Helicobactor pylori, CagA and parietal cells, and ghrelin) tend to be associated with atrophic or inﬂammatory conditions of gastric mucosa, and lack sufficient sensitivity and specificity for accurate GC diagnosis.

MicroRNAs are a large family of post-transcriptional regulators of gene expression that are about 21∼24 nucleotides in length and are abundant in animals, plants and even viruses [5], [6]. They play important roles in the regulation of target genes by binding to their 3′UTR causing targeting deadenylation and destabilization, as well as translational inhibition [7], [8]. Several studies have shown that microRNAs are involved in tumorigenesis and cancer progression and can be stably detected in serum or plasma [9]–[11]. There is evidence showing that some circulating microRNAs originate from cancer tissues, and can be quantitatively measured with established methodology from serum or plasma sample [12]–[14]. Compared to normal individual, distinguishable microRNA expression pattern is observed in GC patients. Therefore, microRNAs gradually show their advantages in the diagnosis and prognosis of GC [15]–[18].

MicroRNA-21 (miR-21) is one of the most frequently studied oncomiRNAs. It has been proved that phosphatase and tensin homologue is the direct target of miR-21 whose expression is elevated in GC tissues and GC derived cell lines [2], [19]–[21]. Chan et al. demonstrated that miR-21 was overexpressed in GC tissues of 92% patients compared to normal counterparts [20]. Taken together these reported studies, it is proposed that miR-21 could serve as an efficient diagnostic marker for GC.

Many research groups have published their findings concerning the application of miR-21 in the diagnosis of GC with varied results. Systematic analysis of these data may be valuable to finally confirm the application potential of miR-21 as biomarker for GC. So the aim of this meta-analysis is to explore the potential value of miR-21 in the diagnosis of GC, which, to the best of our knowledge, has not been previously performed.

## Materials and Methods

### Search Strategy

We searched several relevant international databases (PubMed, Embase, the Cochrane Library, Web of Science, Ovid, SciVerse, Science Direct, Scopus, BioMed Central, Biosis previews) and four Chinese databases (Chinese Biomedical Literature Database-disc, Chinese National Knowledge Infrastructure (CNKI), Technology of Chongqing (VIP), and Wan Fang DATA) up to May 29^th^, 2013. The key words employed for literature retrieval are “microRNA-21µ or “miR-21” or “miRNA-21” or “hsa-miR-21” and “gastric” or “stomach” and “cancer” or “carcinoma” or “tumor” or “neoplasm” or “cancer” or “adenocarcinoma” and “serum” or “sera” or “serums” or “blood” or “plasma”. To obtain additional relevant articles, we scanned conference summaries and reference lists of articles identified in the initial search and even contacted authors to get additional information if necessary.

### Selection of Publications

All publications identified by our search strategy were independently assessed by two reviewers (Z.Y.Z and J.G.W). Any disagreement on controversial study was resolved by fullly discussionto consensus. Studies were included if they meet the following inclusion criteria: (1) the diagnosis of GC was made based on histopathological confirmation, which is widely regarded as the gold standard for GC diagnosis; (2) peripheral blood must have been collected for miR-21 analysis before any treatment; (3) the studies detecting miR-21 concentration in peripheral blood were included; and (4) Studies presenting sufficient data to allow construction of two-by-two tables, and (5) Patients with benign disease or healthy people served as the control group. Additionally, studies exclusion criteria are: (1) duplicate publications; (2) unqualified data; (3) studies with fewer than 30 patients; and (4) having no clear cut-off value in literatures. All of the literatures in line with above criteria are considered to be qualified studies.

### Data Extraction

Data were retrieved from each study independently by two reviewers (Z.Y.Z and J.G.W) including the following characteristics: description of study population (age, gender, clinical stage and node status), study details (first author, year of publication and country of publication), data for two-by-two table (cut-off, sensitivity and specificity) and study design.

### Quality Assessment

The quality of each study was scored independently by two reviewers (Z.Y.Z and J.G.W) with the Quality Assessment of Diagnostic Accuracy Studies (QUADAS) [22] tool which features 14 questions and demonstrated to be an efficient tool for the quality assessment of diagnostic accuracy studies (Table S2). Each question should be answered with “yes”, “no”, or “unclear”. An answer of “yes” will get one score, while the “no” or “unclear” will gain a score of zero with a total score of 14.

### Statistical Analysis

The bivariate meta-analysis model was employed to summarize the sensitivity, specificity, positive likelihood ratio (PLR), negative likelihood ratio (NLR), diagnostic odds ratio (DOR) and generate the bivariate summary receiver operator characteristic (SROC) curve [23]. The bivariate approach preserves the two-level nature of the original data, with independent binomial distributions for true positives and true negatives subject to sensitivity and specificity in each study [23], [24]. Pairs of sensitivity and specificity are jointly analyzed, incorporating any correlation that might exist between these two criteria using a random effects approach. Additionally, explanatory variables can be added to the bivariate model and lead to separate effects on sensitivity and specificity, rather than a net effect on the odds ratio scale as in the sROC approach [24]. Therefore, the bivariate model is considered as a more valid statistical model for diagnostic meta-analysis [25]–[27].While different studies draw different conclusions, this may result from random error or heterogeneity as to differences in clinical or methodological characteristics of studies. Therefore, Chi-square and *I* ^2^ test for heterogeneity were used to assess the heterogeneity in studies. A value of P less than 0.05 and of *I* ^2^ more than 50% indicated the existence of significant heterogeneity [28], [29].

The publication bias of selected studies was assessed using the funnel plot with the Begg’s test and Egger’s test. To detect cut-off threshold effects, the relationship between sensitivity and specificity was evaluated by the Spearman correlation coefficient. Subgroup analyses and sensitivity analysis were also performed if necessary to dissect the heterogeneity. All analyses were performed using stata SE12.0 (Stata Corporation) and Meta-DiSc software [30].

## Results

### Included Studies

**Figure 1:**
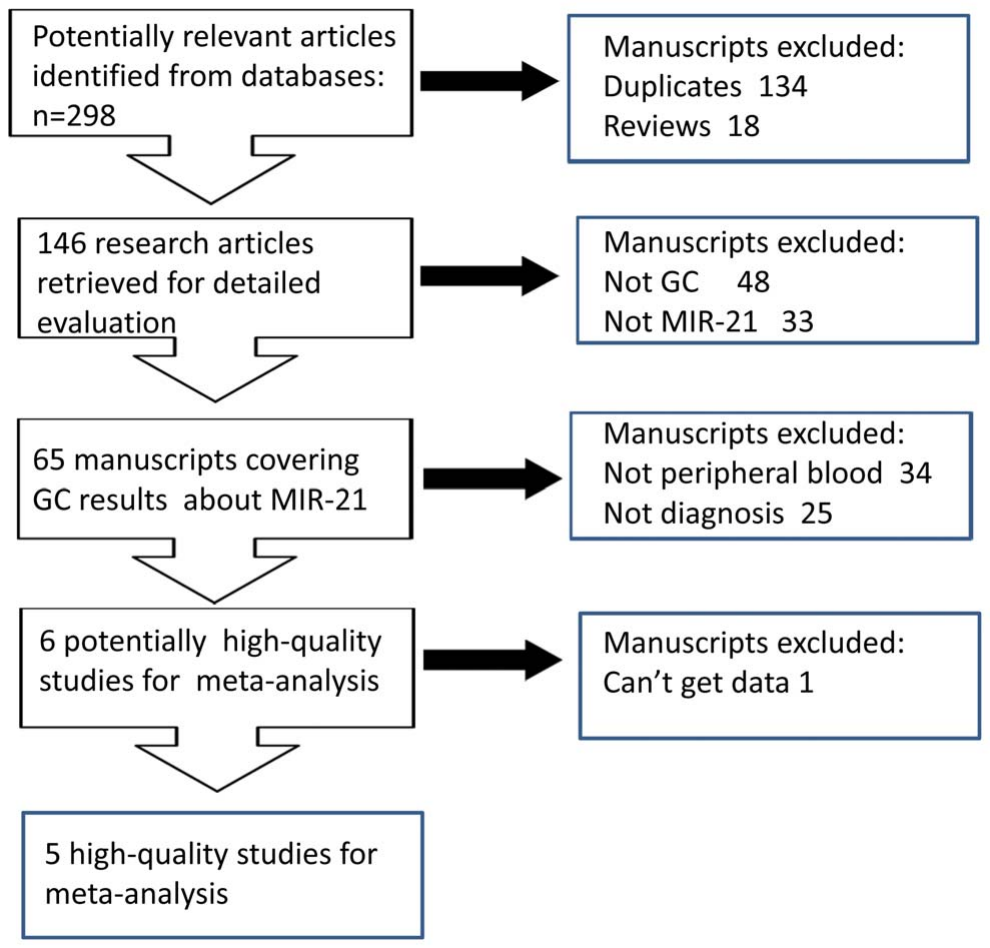
Flow chart of study selection based on the inclusion and exclusion criteria.

The initial search returned a total of 298 manuscripts among which 134duplicated hits and 18 reviews were excluded. The left 146 research articles are subject to the next-step evaluation. And 48 manuscripts were excluded from analysis as the carcinoma was not gastric cancer, leaving 72 studies available for further full text review. After carefully reading the text, 33 manuscripts were excluded as other miRNAs rather than miR-21 were focused. Of the remained 65 manuscripts, samples of 34 studies were not from peripheral blood, 25 studies were not diagnostic research, and one study failed to publish detailed information. Thus, the meta-analysis was performed on the final 5 studies [31]–[35] (Figure 1).

### Study Characteristics and Quality Assessment

In these eligible articles, all of the 251 GC patients had been histopathologically confirmed, which is the gold standard for GC diagnosis. Additionally, the five studies have a well-defined reference standard for stage classification, which includes the usage of definitions established by the AJCC/UICC stage classification (7th edition)[34]–[36], the tumor node metastasis (TNM) staging of the International Union Against Cancer [32], [33] and the IGCC/TMN staging system [31], [37]. And the 184 control individuals are all from healthy volunteers who had never been diagnosed with a malignant tumor. The 5 remaining studies including 251 patients and 184 control samples reported the quantity of miR-21 in peripheral blood. The 5 studies were published from 2010 to 2012. In these studies, miR-21 was detected by reverse transcription Polymerase Chain Reaction (RT-PCR). But in Zheng et al’s study [32] the levels of miR-21 were normalized by the ΔCt method, and in Wang’s study [34] it was normalized by the 2-ΔΔCt method [38]. These results are reflected in Table 1. The 5 studies were scored by QUADAS by two independent reviewers (J.G.W and P.H). The QUADAS scores of analysis show that all studies get a score of 13 indicating high quality (Table S2).

**Table 1:** Summary of studies using miR-21 as a biomarker of GC and study quality assessment.

First author	Year	patients (controls)	QUADASscores	Stage I,II%	Mean or median age	AUC	Cut-off	Se%	Sp%
Tsujiura et al [31]	2010	69(30)	13	73.9	NR	0.673*	0.0373* amol/ul	60.90*	63.33*
Li et al [33]	2012	70(70)	13	33	54	0.794	0.050 amol/ul	74.29	75.71
Zheng et al [32]	2011	53(20)	13	30.2	NR	0.853	7.73 ΔCt	83.01	80.53
Wang et al [34]	2012	30(39)	13	36.7	58	0.81	5.63 2-ΔΔCt	56.7	94.9
Shen et al [35]	2012	29(25)	13	NR	54	0.750*	0.0595*amol/ul	51.70*	92.00*

Note: *Calculated from independent patient data (IPD) QUADAS = quality assessment for studies of diagnostic accuracy. AUC = Area under the curve of a receiver operator curve. NR = not report. Se = sensitivity. Sp = specificity.

### Data Analysis

**Figure 2:**
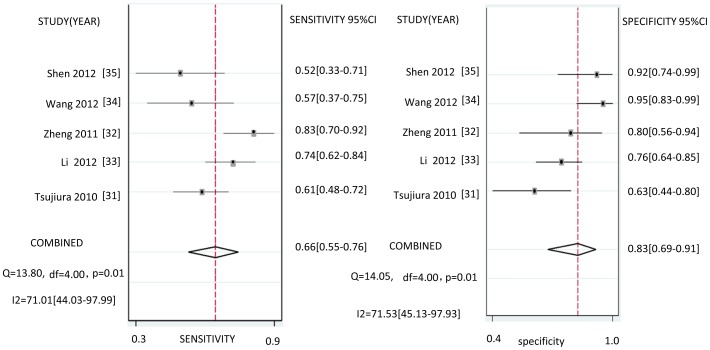
Forest plots of sensitivities and specificities from test accuracy studies of miR-21 in the diagnosis of GC.

Heterogeneity in sensitivity and specificity are observed among the five studies (I2 = 71.01% and I2 = 71.53%), which indicates significant heterogeneity (Figure 2). Therefore, the random effects model was selected in this study. The bivariate meta-analysis shows a pooled sensitivity of miR-21 for the diagnosis of GC of 66% (95%CI, 55%–76%) and a pooled specificity of 83% (95%CI, 69%–91%).

**Figure 3:**
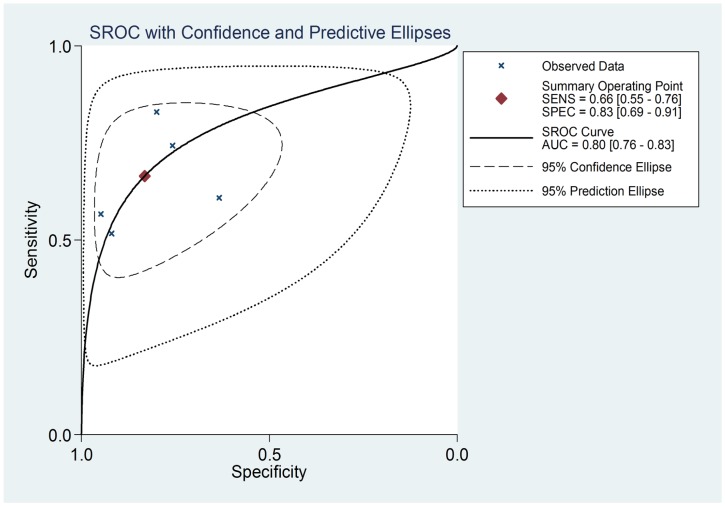
Summary receiver operating characteristic curves for miR-21 in the diagnosis of GC. The smaller region (confidence contour) contains likely combinations of the mean value of sensitivity and specificity. The wider region (prediction contour) demonstrates more uncertainty as to where the likely values of sensitivity and specificity might occur for individual studies.

In the present studies, the combined PLR is 3.95 (95%CI: 2.15–7.24) which indicates that patients with GC have a nearly 4-fold higher chance of being miR-21 test-positive compared with others without GC. In addition, there exists ignoble heterogeneity between PLRs (I2 = 37.83). In respect to NLR, the combined NLR is 0.40 (95%CI: 0.30–0.54) (Figure S1). The heterogeneity analysis shows that the chi-squares valve is 11.63 and I^2^ 65.60%. The SROC curve for the included studies is shown in Figure 3. The AUC is 0.80 (95%CI: 0.76–0.83), and the DOR is 9.8 (95%CI, 4.6–20.8), indicating a moderate diagnostic accuracy. This figure also presents the summary operating point estimate of sensitivity and specificity.

### Publication Bias

**Figure 4:**
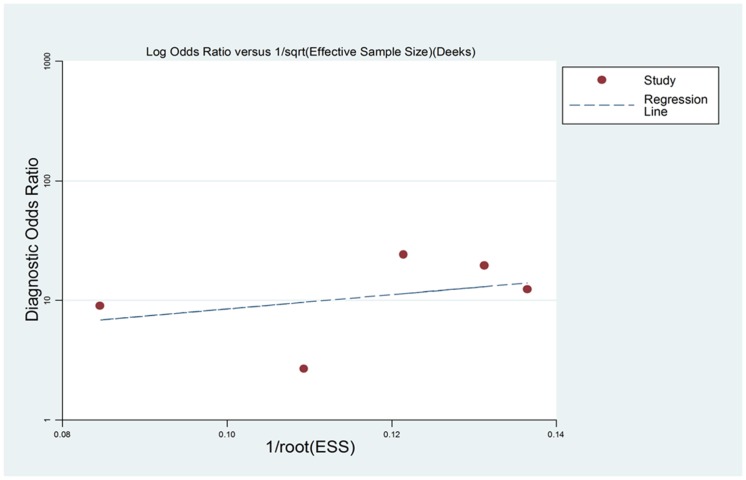
Funnel plot for the assessment of potential bias in miR-21 assays.

To assess the publication bias in this study, funnel plots was used in the meta-analysis. The funnel plot demonstrates a somehow asymmetric curve which can be explained by the limited number of included studies (Figure 4). The P-value of Begg’s test and Egger’s test are 1.0 and 0.361, respectively. Therefore, there is no evidence showing that publication bias exists. However, for the limited number of the articles, whether the publication bias exists in this meta-analysis is still difficult to draw a conclusion.

### Threshold Effect and Heterogeneity

The threshold effect is due to differences of sensitivity and specificity, and Spearman correlation coefficient of sensitivity and specificity is a good approach to evaluate the threshold effect [30]. In this meta-analysis, the Spearman correlation coefficient of sensitivity and 1-specificity was 0.500 with a P value of 0.391 (p>0.05), suggesting that there is no heterogeneity from threshold effect.

The *I* ^2^ of heterogeneity test is 71.38%, indicating moderate heterogeneity. Initially, we consider that the test method, publication country, number of patient and the representation of the participants (stage I, II %) may contribute to the heterogeneity (Table S1). However, meta-regression analysis indicates that above variables were not the sources of heterogeneity for this study. Sensitivity analysis finds that the meta-analysis is vigorously influenced obviously by individual study. For example, if the data of Tsujiura et al. [31] was removed, the analysis results changed significantly without obvious heterogeneity between remained studies (chi-squared = 1.81 (p = 0.612) and I^2^ = 0.0%). The pooled DOR of the 4 homogeneity studies were 12.319.

## Discussion

As to patients with GC, low early diagnosed rate and low 5-year survival rate are two key factors that influence the prognosis of this disease and largely impair their health condition [1], [3]. To the best of our knowledge, there is no effective diagnostic biomarker for GC with desirable sensitivity and specificity. Accordingly, diagnosis of GC is based on by histological examination but it only works at the advanced stage of disease when the effectiveness of medical care interference is compromised. More and more attention has been paid to the improvement of early diagnosis of GC [4], [39]–[43]. Recent studies have brought an explosion of new diagnostic markers of GC including miR-21. To evaluate the diagnostic and clinical valve of miR-21as a serological marker, we conducted this meta-analysis to provide a comprehensive and up-to-date analysis of the feasibility and accuracy of miR-21 for the diagnosis of GC. As far as we know, this is the first meta-analysis about the diagnostic value of miR-21for GC.

A plasma-based diagnostic test is inherently more attractive in a solid-organ malignancy diagnosis. In this meta-analysis, we show that the pooled sensitivity and specificity are 0.665 (95% CI: 0.550–0.763) and 0.831 (95%CI: 0.694–0.915) respectively. Thus, miR-21 enjoys it has higher sensitivity and specificity compared to conventional serum biomarker such as CEA (sensitivity of 26.8%) andCA19-9 (sensitivity of 33.8%) [44], [45]. it has higher sensitivity and specificity in effectively diagnosing of GC. Glas et al. [46] found that the diagnostic odds ratio (DOR) combines the strengths of sensitivity and specificity as prevalence in dependent indicators and has the advantage of accuracy over a single indicator. The value of DOR ranges from 0 to infinity with higher values indicating better discriminatory test performance [46]. The DOR value of 9.791 indicates that the miR-21 could be a useful biomarker for GC patients’ diagnosis. SROC is usually used to summarize overall test performance, and AUC is calculated to evaluate accuracy of the selected indicator. To demonstrate excellent accuracy, the valve of AUC should be more than 0.97. An AUC of 0.93 to 0.96 is considered to be very good and 0.75 to 0.92 is good. However, a value of less than 0.75 can be still reasonable, while the test will have obvious deficiency in its diagnostic accuracy, approaching a random test [47], [48]. In these studies, we show that miR-21 demonstrates good accuracy in the diagnosis of gastric cancer, with an area under the ROC curve of 0.80. Overall, although the sensitivity is compromised, miR-21 has a good specificity in the diagnosis of GC.

Heterogeneity is a potential problem when interpreting the results for all meta-analysis. One of the primary causes of heterogeneity in test accuracy studies is threshold effect, which arises when differences in sensitivities and specificities occur due to different cut-offs or thresholds used in different studies to define a positive or negative test result. As different cut-off values were used among the 5 studies, we used the Spearman correlation coefficient to analyze the threshold effect. The Spearman correlation coefficient of sensitivity and 1-specificity is 0.500 (*p* = 0.391<0.05), which indicates that there is no heterogeneity from threshold effects. Although the detecting methods for miR-21 are all based on reverse transcriptional PCR (RT-PCR) and real-time quantification PCR (qPCR), there are no unified primers and no reference miRNAs for qPCR analysis. Therefore, different laboratories take different measures to quantify the miR-21, which may contribute to sources of heterogeneity. With regard to this, we performed a meta-regression analysis to assess the contribution of factors above and find that none of these variables are the sources of heterogeneity. However, sensitivity analysis determines that the heterogeneity is from Tsujiuria et al’s study [31] for when it was removed, there shows no heterogeneity among remained four studies (chi-squared = 1.81 (p = 0.612) and I^2^ = 0.0%).Thus, we conclude that the heterogeneity at least partially come from the publication area bias.

Although we tried to avoid the biases in the process of meta-analysis, there were still several limitations to our study. Firstly, miR-21 as a novel marker in GC diagnosis just looms in recent years, and still limited research work was done on the diagnosis value of miR-21. So, the study size obtained in this meta-analysis is relatively small. Secondly, although we have tried our best to cover all the involved literatures by a comprehensive method without language restriction, we may still miss some of them during the screen process. Thirdly, despite of our best efforts such as by searching other related references, e-mail, and fax to all authors, we could not acquire the independent patient data(IPD) of Wang et al’s [34] study for further study. Fourthly, there is no evidence that publication bias exists (p = 0.541) by funnel plots, however, these studies are either from China or Japan indicating that the publication area bias may still exist. The reason for this may be that miR-21 is a new biomarker for gastric cancer, and more prospective studies about the diagnostic value of miR-21 for GC are needed in future. Furthermore, the highest mortality rates of GC are estimated in Eastern Asia [49], this may inspire more researchers to study the early diagnosis and treatment of GC. In conclusion, despite of the limitations mentioned above, the current evidence suggests that miR-21 has potential diagnostic value with good specificity and considerable moderate sensitivity for GC. Larger-scale prospective studies are needed in future. In addition, how to improve the accuracy should be considered and novel GC markers with more pronounced accuracy remain to be explored in future.

## Supporting Information

Figure S1 **Forest plot of PLR and DLR from test accuracy studies of miR-21 in the diagnosis of GC.**
(TIF)

Table S1 Detail information of meta-regression and subgroup analysis.
(TIF)

Table S2 The Quality Assessment of Diagnostic Accuracy Studies (QUADAS).
(TIF)

Checklist S1 **PRISMA checklist.**
(DOC)

## References

[1] Society AAC (2011) Global Cancer Facts & Figures 2nd Edition. American Cancer Society.

[2] Meng F, Henson R, Lang M, Wehbe H, Maheshwari S, et al (2006) Involvement of human micro-RNA in growth and response to chemotherapy in human cholangiocarcinoma cell lines. Gastroenterology 130: 2113–2129. 16762633 10.1053/j.gastro.2006.02.057

[3] Garcia M, Jemal A (2011) Global Cancer Facts and Figures 2011. Atlanta, GA: American Cancer Society.

[4] Tan YK, Fielding JW (2006) Early diagnosis of early gastric cancer. European journal of gastroenterology & hepatology 18: 821–829. 16825897 10.1097/00042737-200608000-00004

[5] Flynt AS, Lai EC (2008) Biological principles of microRNA-mediated regulation: shared themes amid diversity. Nat Rev Genet 9: 831–842. 18852696 10.1038/nrg2455 PMC2729318

[6] Carthew RW, Sontheimer EJ (2009) Origins and Mechanisms of miRNAs and siRNAs. Cell 136: 642–655. 19239886 10.1016/j.cell.2009.01.035 PMC2675692

[7] Lewis BP, Burge CB, Bartel DP (2005) Conserved seed pairing, often flanked by adenosines, indicates that thousands of human genes are microRNA targets. Cell 120: 15–20. 15652477 10.1016/j.cell.2004.12.035

[8] Krol J, Loedige I, Filipowicz W (2010) The widespread regulation of microRNA biogenesis, function and decay. Nat Rev Genet 11: 597–610. 20661255 10.1038/nrg2843

[9] Calin GA, Croce CM (2006) MicroRNA signatures in human cancers. Nature reviews Cancer 6: 857–866. 17060945 10.1038/nrc1997

[10] Chen X, Ba Y, Ma L, Cai X, Yin Y, et al (2008) Characterization of microRNAs in serum: a novel class of biomarkers for diagnosis of cancer and other diseases. Cell Res 18: 997–1006. 18766170 10.1038/cr.2008.282

[11] Filipowicz W, Bhattacharyya SN, Sonenberg N (2008) Mechanisms of post-transcriptional regulation by microRNAs: are the answers in sight? Nat Rev Genet 9: 102–114. 18197166 10.1038/nrg2290

[12] Mitchell PS, Parkin RK, Kroh EM, Fritz BR, Wyman SK, et al (2008) Circulating microRNAs as stable blood-based markers for cancer detection. Proceedings of the National Academy of Sciences of the United States of America 105: 10513–10518. 18663219 10.1073/pnas.0804549105 PMC2492472

[13] Zamore PD, Haley B (2005) Ribo-gnome: the big world of small RNAs. Science 309: 1519–1524. 16141061 10.1126/science.1111444

[14] Calin GA, Sevignani C, Dumitru CD, Hyslop T, Noch E, et al (2004) Human microRNA genes are frequently located at fragile sites and genomic regions involved in cancers. Proceedings of the National Academy of Sciences of the United States of America 101: 2999–3004. 14973191 10.1073/pnas.0307323101 PMC365734

[15] Jiang Z, Guo J, Xiao B, Miao Y, Huang R, et al (2010) Increased expression of miR-421 in human gastric carcinoma and its clinical association. J Gastroenterol 45: 17–23. 19802518 10.1007/s00535-009-0135-6

[16] Zhang Y, Guo J, Li D, Xiao B, Miao Y, et al (2010) Down-regulation of miR-31 expression in gastric cancer tissues and its clinical significance. Med Oncol 27: 685–689. 19598010 10.1007/s12032-009-9269-x

[17] Zhang B, Pan X, Cobb GP, Anderson TA (2007) microRNAs as oncogenes and tumor suppressors. Dev Biol 302: 1–12. 16989803 10.1016/j.ydbio.2006.08.028

[18] Cui L, Zhang X, Ye G, Zheng T, Song H, et al (2013) Gastric juice MicroRNAs as potential biomarkers for the screening of gastric cancer. Cancer 119: 1618–1626. 23335180 10.1002/cncr.27903

[19] Yamanaka Y, Tagawa H, Takahashi N, Watanabe A, Guo YM, et al (2009) Aberrant overexpression of microRNAs activate AKT signaling via down-regulation of tumor suppressors in natural killer-cell lymphoma/leukemia. Blood 114: 3265–3275. 19641183 10.1182/blood-2009-06-222794

[20] Chan S-H, Wu C-W, Li AF-Y, Chi C-W, Lin W-C (2008) miR-21 microRNA expression in human gastric carcinomas and its clinical association. Anticancer research 28: 907–911. 18507035

[21] Zhang Z, Li Z, Gao C, Chen P, Chen J, et al (2008) miR-21 plays a pivotal role in gastric cancer pathogenesis and progression. Laboratory Investigation 88: 1358–1366. 18794849 10.1038/labinvest.2008.94

[22] Whiting P, Rutjes AW, Reitsma JB, Bossuyt PM, Kleijnen J (2003) The development of QUADAS: a tool for the quality assessment of studies of diagnostic accuracy included in systematic reviews. BMC Medical Research Methodology 3: 25. 14606960 10.1186/1471-2288-3-25 PMC305345

[23] Mitchell AJ, Vaze A, Rao S (2009) Clinical diagnosis of depression in primary care: a meta-analysis. The Lancet 374: 609–619. 10.1016/S0140-6736(09)60879-5 19640579

[24] Reitsma JB, Glas AS, Rutjes AW, Scholten RJ, Bossuyt PM, et al (2005) Bivariate analysis of sensitivity and specificity produces informative summary measures in diagnostic reviews. Journal of clinical epidemiology 58: 982–990. 16168343 10.1016/j.jclinepi.2005.02.022

[25] Harbord RM, Deeks JJ, Egger M, Whiting P, Sterne JA (2007) A unification of models for meta-analysis of diagnostic accuracy studies. Biostatistics 8: 239–251. 16698768 10.1093/biostatistics/kxl004

[26] Harbord RM, Whiting P, Sterne J, Egger M, Deeks JJ, et al (2008) An empirical comparison of methods for meta-analysis of diagnostic accuracy showed hierarchical models are necessary. Journal of clinical epidemiology 61: 1095–1103. 19208372 10.1016/j.jclinepi.2007.09.013

[27] Leeflang MM, Deeks JJ, Gatsonis C, Bossuyt PM (2008) Systematic reviews of diagnostic test accuracy. Annals of internal medicine 149: 889–897. 19075208 10.7326/0003-4819-149-12-200812160-00008 PMC2956514

[28] Higgins J, Thompson SG, Deeks JJ, Altman DG (2003) Measuring inconsistency in meta-analyses. Bmj 327: 557–560. 12958120 10.1136/bmj.327.7414.557 PMC192859

[29] Dinnes J, Deeks J, Kirby J, Roderick P (2005) A methodological review of how heterogeneity has been examined in systematic reviews of diagnostic test accuracy. Health Technology Assessment 9: 1–113. 10.3310/hta9120 15774235

[30] Zamora J, Abraira V, Muriel A, Khan K, Coomarasamy A (2006) Meta-DiSc: a software for meta-analysis of test accuracy data. BMC medical research methodology 6: 31. 16836745 10.1186/1471-2288-6-31 PMC1552081

[31] Tsujiura M, Ichikawa D, Komatsu S, Shiozaki A, Takeshita H, et al (2010) Circulating microRNAs in plasma of patients with gastric cancers. Br J Cancer 102: 1174–1179. 20234369 10.1038/sj.bjc.6605608 PMC2853097

[32] Zheng Y, Cui L, Sun W, Zhou H, Yuan X, et al (2011) MicroRNA-21 is a new marker of circulating tumor cells in gastric cancer patients. Cancer Biomark 10: 71–77. 22430134 10.3233/CBM-2011-0231 PMC13016254

[33] Li BS, Zhao YL, Guo G, Li W, Zhu ED, et al (2012) Plasma microRNAs, miR-223, miR-21 and miR-218, as novel potential biomarkers for gastric cancer detection. PLoS One 7: e41629. 22860003 10.1371/journal.pone.0041629 PMC3408505

[34] Wang B, Zhang Q (2012) The expression and clinical significance of circulating microRNA-21 in serum of five solid tumors. J Cancer Res Clin Oncol 138: 1659–1666. 22638884 10.1007/s00432-012-1244-9 PMC11824721

[35] Shen J, Feng C, Hao B, Mei L (2012) Application of miR-21 and let-7a in serum for non-invasive diagnosis of gastric cancer and evaluation of surgery results. Journal of Zhengzhou University(Medical Sciences) 47: 722–725.

[36] Edge SB, Compton CC (2010) The American Joint Committee on Cancer: the 7th edition of the AJCC cancer staging manual and the future of TNM. Annals of surgical oncology 17: 1471–1474. 20180029 10.1245/s10434-010-0985-4

[37] Sobin L, Wittekind C (2002) Intenational Union Against Cancer (UICC). TNM Classification of Malignant Tumors 6th edn John Wiley & Sons. Inc: New York.

[38] Schmittgen TD, Livak KJ (2008) Analyzing real-time PCR data by the comparative CT method. Nature protocols 3: 1101–1108. 18546601 10.1038/nprot.2008.73

[39] Nobili S, Bruno L, Landini I, Napoli C, Bechi P, et al (2011) Genomic and genetic alterations influence the progression of gastric cancer. World journal of gastroenterology 17: 290–299. 21253387 10.3748/wjg.v17.i3.290 PMC3022288

[40] Yamashita K, Sakuramoto S, Watanabe M (2011) Genomic and epigenetic profiles of gastric cancer: potential diagnostic and therapeutic applications. Surgery today 41: 24–38. 21191688 10.1007/s00595-010-4370-5

[41] Ye T, Chen Y, Fang J (2010) DNA methylation biomarkers in serum for gastric cancer screening. Mini Reviews in Medicinal Chemistry 10: 1034–1038. 20716059 10.2174/1389557511009011034

[42] Sapari NS, Loh M, Vaithilingam A, Soong R (2012) Clinical potential of DNA methylation in gastric cancer: a meta-analysis. PLoS One 7: e36275. 22558417 10.1371/journal.pone.0036275 PMC3338684

[43] Shiota S, Matsunari O, Watada M, Yamaoka Y (2010) Serum Helicobacter pylori CagA antibody as a biomarker for gastric cancer in east-Asian countries. Future Microbiol 5: 1885–1893. 21155667 10.2217/fmb.10.135 PMC3044821

[44] Zhu YB, Ge SH, Zhang LH, Wang XH, Xing XF, et al (2012) [Clinical value of serum CEA, CA19-9, CA72-4 and CA242 in the diagnosis and prognosis of gastric cancer]. Zhonghua Wei Chang Wai Ke Za Zhi 15: 161–164. 22368025

[45] Li Y, Yang Y, Lu M, Shen L (2011) Predictive value of serum CEA, CA19-9 and CA72.4 in early diagnosis of recurrence after radical resection of gastric cancer. Hepato-gastroenterology 58: 2166–2170. 22024091 10.5754/hge11753

[46] Glas AS, Lijmer JG, Prins MH, Bonsel GJ, Bossuyt PMM (2003) The diagnostic odds ratio: a single indicator of test performance. Journal of Clinical Epidemiology 56: 1129–1135. 14615004 10.1016/s0895-4356(03)00177-x

[47] Jones CM, Athanasiou T (2005) Summary receiver operating characteristic curve analysis techniques in the evaluation of diagnostic tests. Ann Thorac Surg 79: 16–20. 15620907 10.1016/j.athoracsur.2004.09.040

[48] Walter SD (2002) Properties of the summary receiver operating characteristic (SROC) curve for diagnostic test data. Stat Med 21: 1237–1256. 12111876 10.1002/sim.1099

[49] Ferlay J, Shin HR, Bray F, Forman D, Mathers C, et al (2010) Estimates of worldwide burden of cancer in 2008: GLOBOCAN 2008. International journal of cancer 127: 2893–2917. 21351269 10.1002/ijc.25516

