# Supplementary figures and images for: Potential Role of microRNA-21 in the Diagnosis of Gastric Cancer: A Meta-Analysis
Source: PLoS One. 2013 Sep 4;8(9):e73278. doi: 10.1371/journal.pone.0073278 (PMC3762732; doi:10.1371/journal.pone.0073278)

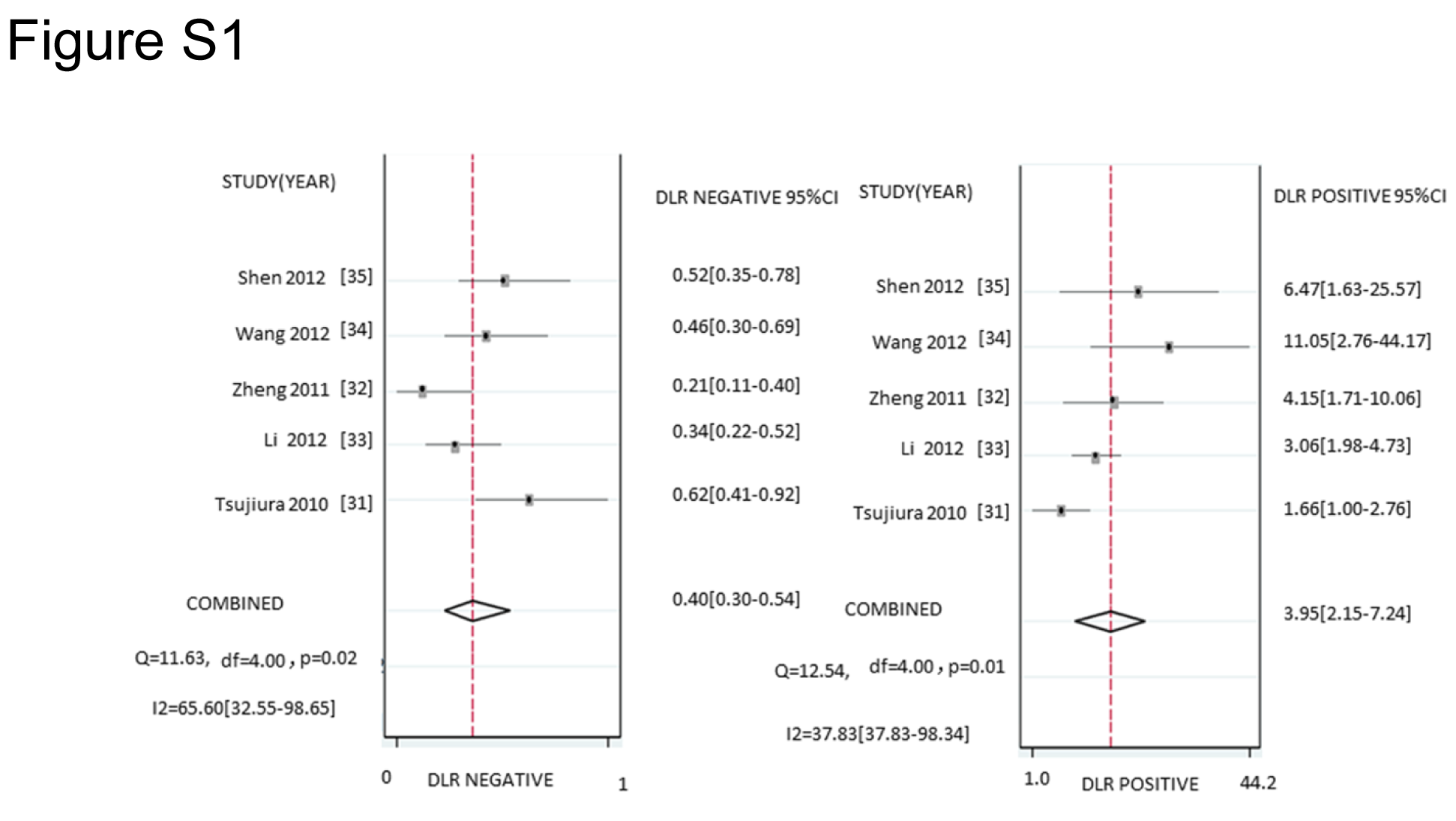

Supplement: Figure S1 — Forest plot of PLR and DLR from test accuracy studies of miR-21 in the diagnosis of GC. (TIF) [file pone.0073278.s001.tif]

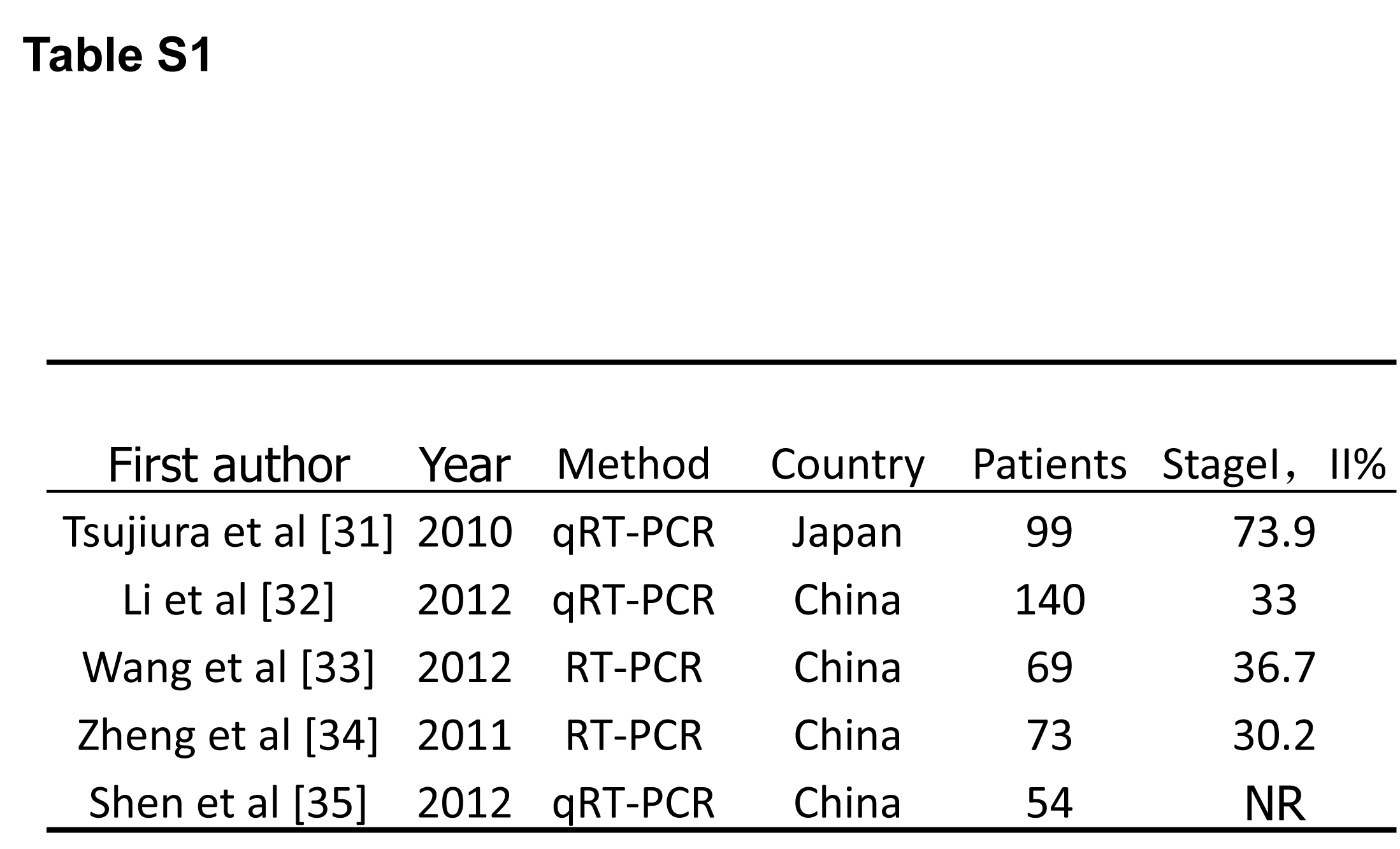

Supplement: Table S1 — Detail information of meta-regression and subgroup analysis. (TIF) [file pone.0073278.s002.tif]

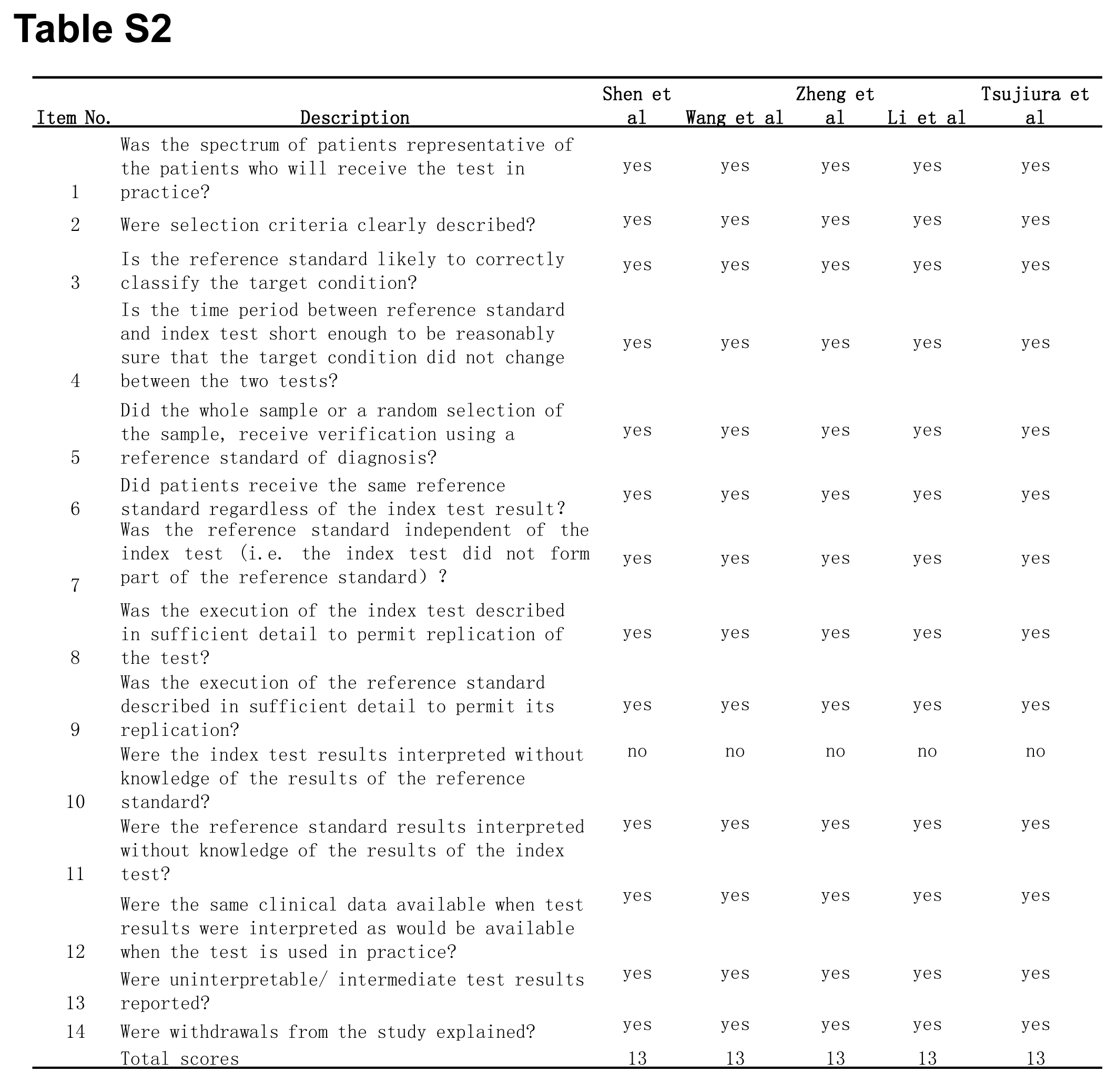

Supplement: Table S2 — The Quality Assessment of Diagnostic Accuracy Studies (QUADAS). (TIF) [file pone.0073278.s003.tif]
