# Supplementary material for: Potential Role of microRNA-21 in the Diagnosis of Gastric Cancer: A Meta-Analysis
Source: PLoS One. 2013 Sep 4;8(9):e73278. doi: 10.1371/journal.pone.0073278 (PMC3762732; doi:10.1371/journal.pone.0073278)
Supplement: Checklist S1 — PRISMA checklist. (DOC) [file pone.0073278.s004.doc]

| **Section/topic** | **#** | **Checklist item** | **Reported on page #** |
| --- | --- | --- | --- |
| **TITLE** | | |  |
| Title | 1 | Potential role of microRNA-21 in the diagnosis of gastric cancer: a meta-analysis | 1 |
| **ABSTRACT** | | |  |
| Structured summary | 2 | **Abstract**  **Introduction:** Accumulating evidences indicate that microRNA-21(miR-21) show significant high concentration in plasma of gastric cancer (GC) patients compared to normal individuals, suggesting that it may be a useful novel diagnostic biomarker for gastric cancer. Therefore, we aimed to assess the potential diagnostic value of miR-21 for gastric cancer in this study.  **Methods:** Literature database including PubMed, Embase, the Cochrane Library, Web of Science, Ovid, SciVerse, Science Direct, Scopus, BioMed Central, Biosis previews，Chinese Biomedical Literature Database (CBM), Chinese National Knowledge Infrastructure (CNKI), Technology of Chongqing (VIP), and Wan Fang DATA were searched for publications concerning the diagnostic value of miR-21 for GC without language restriction. . The quality of each study was scored with the Quality Assessment of Diagnostic Accuracy Studies (QUADAS). Then, data were retrieved from any qualified article hits and subject to meta-analysis. Receiver operating characteristic curves (ROC) were used to check the overall test performance. Evidence of heterogeneity was evaluated using the Chi-square and *I*^2^ test.  Results: Five studies with a total 251 GC patients and 184 control individuals were included in this meta-analysis. All of the included studies are of high quality (QUADAS score$13). The summary estimates revealed that the pooled sensitivity is 66.5% (95% confidence interval (CI): 55.0%-76.3%) and the specificity is 83.1% (95% CI: 69.4%-91.5%). In addition, the area under the summary ROC curve (AUC) is 0.80.  **Conclusion:** The current evidence suggests that miR-21 has potential diagnostic value with a moderate sensitivity and specificity for GC. More prospective studies on the diagnostic value of miR-21 for GC are needed in the future. | 1 |
| **INTRODUCTION** | | |  |
| Rationale | 3 | Numerous groups have now published their experience with the mir-21 test in the diagnosis of gastric cancer, and varying results raise concerns about the mir-21 as a biomarker in the early detection of gastric cancer. | 2 |
| Objectives | 4 | So own aim is to explore the potential value of mir-21 in the diagnosis of GC by this meta-analysis, which, to our knowledge, has not previously been performed. | 2 |
| **METHODS** | | |  |
| Protocol and registration | 5 | There is no review protocol existed. |  |
| Eligibility criteria | 6 | (1) the diagnosis of GC was made based on histopathological conﬁrmation, which is widely regarded as the gold standard for GC diagnosis; (2) peripheral blood must have been collected for miR-21 analysis before any treatment; (3) the studies detecting miR-21 concentration in peripheral blood were included; and (4) Studies presenting sufﬁcient data to allow construction of two-by-two tables, and (5) Patients with benign disease or healthy people served as the control group. | 3 |
| Information sources | 7 | international databases (PubMed, Embase, the Cochrane Library, Web of Science, Ovid, SciVerse, Science Direct, Scopus, BioMed Central, Biosis previews) and four Chinese databases (Chinese Biomedical Literature Database-disc, Chinese National Knowledge Infrastructure (CNKI),Technology of Chongqing (VIP), and Wan Fang DATA) | 2 |
| Search | 8 | The key words employed for literature retrieval are “microRNA-21” or “miR-21” or “miRNA-21” or “hsa-miR-21” and “gastric” or “stomach” and “cancer” or “carcinoma” or “tumor” or “neoplasm” or “cancer” or “adenocarcinoma” and “serum” or “sera” or “serums” or “blood” or “plasma”. | 2-3 |
| Study selection | 9 | All publications identified by our search strategy were independently assessed by two reviewers. Disagreement about study selection was resolved by discussion and consensus. | 3 |
| Data collection process | 10 | Two reviewers independently extracted data in every study to obtain information. | 3 |
| Data items | 11 | Two-by-two table, cut-off value, sensitivity, specificity, study design, age, gender, clinical stage, node status, first author, year of publication, country of publication. | 3 |
| Risk of bias in individual studies | 12 | The publication bias of selected studies was assessed using the funnel plot, meanwhile the Begg’s test and Egger’s test were used to evaluate publication bias statistically. | 3 |
| Summary measures | 13 | The diagnostic meta-analysis was performed using a bivariate meta-analysis model to summarize the sensitivity, speciﬁcity, positive likelihood ratio (PLR), negative likelihood ratio (NLR), diagnostic odds ratio (DOR) and constructed a bivariate summary receiver operator characteristic (SROC) curve. | 3 |
| Synthesis of results | 14 | Analyses were performed using stata SE12.0 and Meta-DiSc software. Pooled sensitivity and specificity, PLR, NLR, SROC and DOR were calculated. Consistency was done by Q-test and I^2^ test. | 3 |

Page 1 of 2

| **Section/topic** | **#** | **Checklist item** | **Reported on page #** |
| --- | --- | --- | --- |
| Risk of bias across studies | 15 | publication bias, selective reporting within studies | 3 |
| Additional analyses | 16 | Meta-regression, sensitivity and subgroup analyses. | 3-4 |
| **RESULTS** | | |  |
| Study selection | 17 | The initial search returned a total of 298 manuscripts among which 134duplicated hits and 18 reviews were excluded. The left 146 research articles are subject to the next-step evaluation. And 48 manuscripts were excluded from analysis as the carcinoma was not gastric cancer, leaving 72 studies available for further full text review. After carefully reading the text, 33 manuscripts were excluded as other miRNAs rather than miR-21 were focused. Of the remained 65 manuscripts, samples of 34 studies were not from peripheral blood, 25 studies were not diagnostic research, and one study failed to publish detailed information. Thus, the meta-analysis was performed on the final 5 studies. | 4 |
| Study characteristics | 18 | age, gender, clinical stage, node status, first author, year of publication, country of publication | 4 |
| Risk of bias within studies | 19 | Funnel plots about mir-21 was used to show the publication bias in the meta-analysis. The result of the test for publication bias was not significant (p=0.541), the shape of funnel plots revealed a bit of asymmetry in some level due to the limited number of articles we selected(Figure S2). Then, Begg’s test and Egger’s test were performed and the P value was 1.0 and 0.361, respectively. There was no evidence that publication bias existed. However, for the limited number of the articles, whether the publication bias existed in this meta-analysis is difficult to draw a conclusion. | 5 |
| Results of individual studies | 20 | Heterogeneity in sensitivity and specificity were observed among the four studies (Q-test=13.80, I2=71.01% and Q-test=14.05, I2=71.53%), which indicated significant heterogeneity for these included studies (Figure 2). The existence of signiﬁcant heterogeneity occurred in the 5 studies, thus the random effects model approach was selected in this study. The bivariate meta-analysis resulted in a pooled sensitivity of mir-21 for the diagnosis of GC of 66% (95%CI, 55–76%) and a pooled speciﬁcity of 83% (95%CI, 69–91%). | 4 |
| Synthesis of results | 21 | Sensitivity: 66 %( 95%CI, 55–76%, Q-test=13.80, I^2^=71.01%) and speciﬁcity: 83 %( 95%CI, 69–91%, Q-test=14.05, I^2^=71.53%). PLR: 3.95(95%CI: 2.15–7.24, Q=12.54, I^2^=37.83), NLR: 0.40 (95%CI: 0.30–0.54, Q = 11.63and I^2^= 65.60%). AUC: 0.80(95%CI: 0.76–0.83), and the Diagnostic Odds Ratio: 9.8(95% CI, 4.6–20.8). | 4 |
| Risk of bias across studies | 22 | Begg’s test and Egger’s test were performed and the P value was 1.0 and 0.361. | 4 |
| Additional analysis | 23 | Sensitivity analysis determined that the meta-analysis was influenced obviously by individual study. When Tsujiura, M’s paper was removed, the data analysis results changed significantly, and there was no heterogeneity between remained studies (chi-squared = 1.81 (p = 0.612) and I2= 0.0%). | 5 |
| **DISCUSSION** | | |  |
| Summary of evidence | 24 | In this meta-analysis, the pooled sensitivity and speciﬁcity were 0.665 (95% CI: 0.550-0.763) and 0.831(95%CI: 0.694-0.915) respectively. The results reported showed that plasma mir-21 had good accuracy in the diagnosis of gastric cancer, with an area under the ROC curve of 0.80. Overall, we considered that the results of mir-21 in GC were satisfactory. | 6 |
| Limitations | 25 | Although we tried to avoid the bias in the process of meta-analysis, there were several limitations to our study. Firstly, mir-21 as a novel marker in GC patients just was researched in recent years, and the diagnosis value of mir-21 was explored rarer. So study size obtained in this meta-analysis was not satisfied. Secondly, although we searched the studies published in Chinese and English, we did not search other languages or unpublished data. For these reasons, some other languages and unpublished data might not have been included in our study, which may have influences on the pooled results. Thirdly, despite of our best efforts such as by searching other related references, e-mail, and fax to all authors, we could not acquire the independent patient data(IPD) of Wang’s study for further study. Fourthly, all the countries of included studies are from Asia, and all samples were collected from Chinese or Japanese. | 7 |
| Conclusions | 26 | In conclusion, despite of the limitations mentioned above, the current evidence suggests that miR-21 has potential diagnostic value with good specificity and considerable moderate sensitivity for GC. Larger-scale prospective studies are needed in future. In addition, how to improve the accuracy should be considered and novel GC markers with more pronounced accuracy remain to be explored in future. | 7 |
| **FUNDING** | | |  |
| Funding | 27 | No funding. |  |

*From:*  Moher D, Liberati A, Tetzlaff J, Altman DG, The PRISMA Group (2009). Preferred Reporting Items for Systematic Reviews and Meta-Analyses: The PRISMA Statement. PLoS Med 6(6): e1000097. doi:10.1371/journal.pmed1000097

For more information, visit: **www.prisma-statement.org**.

Page 2 of 2
